# Machine learning-based combination of the central vein sign, cortical lesions and paramagnetic rim lesions: a web-based tool for the diagnosis of multiple sclerosis

**DOI:** 10.1093/braincomms/fcag079

**Published:** 2026-03-11

**Authors:** Maxence Wynen, Colin Vanden Bulcke, Serena Borrelli, Pedro M Gordaliza, Anna Stölting, François Guisset, Clément Cordier, Maria Sofia Martire, Agnese Tamanti, Benoit Macq, Pascal Sati, Massimo Filippi, Massimiliano Calabrese, Martina Absinta, Daniel S Reich, Meritxell Bach Cuadra, Pietro Maggi

**Affiliations:** ICTEAM Institute, Université Catholique de Louvain, 1348 Louvain-la-Neuve, Belgium; Neuroinflammation Imaging Lab (NIL), Université Catholique de Louvain, 1200 Brussels, Belgium; ICTEAM Institute, Université Catholique de Louvain, 1348 Louvain-la-Neuve, Belgium; Neuroinflammation Imaging Lab (NIL), Université Catholique de Louvain, 1200 Brussels, Belgium; Department of Neurology, Cliniques Universitaires Saint-Luc (CUSL), Université Catholique de Louvain, 1200 Brussels, Belgium; Neuroinflammation Imaging Lab (NIL), Université Catholique de Louvain, 1200 Brussels, Belgium; Department of Neurology, Hôpital Erasme, Hôpital Universitaire de Bruxelles, Université Libre de Bruxelles, 1070 Brussels, Belgium; CIBM Center for Biomedical Imaging, CH-1015 Lausanne, Switzerland; Radiology Department, Lausanne University Hospital (CHUV) and University of Lausanne, CH-1011 Lausanne, Switzerland; Neuroinflammation Imaging Lab (NIL), Université Catholique de Louvain, 1200 Brussels, Belgium; Neuroinflammation Imaging Lab (NIL), Université Catholique de Louvain, 1200 Brussels, Belgium; ICTEAM Institute, Université Catholique de Louvain, 1348 Louvain-la-Neuve, Belgium; Neurology Unit, IRCCS San Raffaele Hospital, 20132 Milan, Italy; Department of Neurosciences and Biomedicine and Movement, The Multiple Sclerosis Center of University Hospital of Verona, 37129 Verona, Italy; ICTEAM Institute, Université Catholique de Louvain, 1348 Louvain-la-Neuve, Belgium; Department of Neurology, Cedars-Sinai Medical Center, 90048 Los Angeles, CA, USA; Neurology Unit, IRCCS San Raffaele Hospital, 20132 Milan, Italy; Vita-Salute San Raffaele University, Milan, Italy; Neuroimaging Research Unit, Division of Neuroscience, IRCCS San Raffaele Scientific Institute, 20132 Milan, Italy; Department of Neurosciences and Biomedicine and Movement, The Multiple Sclerosis Center of University Hospital of Verona, 37129 Verona, Italy; Experimental Neuropathology Lab, IRCCS Humanitas Research Institute, 20132 Milan, Italy; Department of Biomedical Sciences, Humanitas University, 20072 Milan, Italy; Translational Neuroradiology Section, National Institute of Neurological Disorders and Stroke (NINDS), National Institutes of Health (NIH), 20892 Bethesda, MD, USA; CIBM Center for Biomedical Imaging, CH-1015 Lausanne, Switzerland; Radiology Department, Lausanne University Hospital (CHUV) and University of Lausanne, CH-1011 Lausanne, Switzerland; Neuroinflammation Imaging Lab (NIL), Université Catholique de Louvain, 1200 Brussels, Belgium; Department of Neurology, Cliniques Universitaires Saint-Luc (CUSL), Université Catholique de Louvain, 1200 Brussels, Belgium

**Keywords:** multiple sclerosis, machine learning, diagnosis, MRI, computer-aided diagnosis

## Abstract

Multiple sclerosis diagnostic criteria lack optimal specificity, leading to potential misdiagnosis. Advanced magnetic resonance imaging (MRI) biomarkers like the central vein sign, cortical lesions and paramagnetic rim lesions are highly specific to multiple sclerosis and could potentially improve diagnostic accuracy. In this study, we applied machine learning techniques to a retrospective, multicentric dataset of 322 multiple sclerosis/multiple sclerosis-mimic (204/118) and 84 prodromal multiple sclerosis/non-multiple sclerosis (43/41) adult patients, incorporating the central vein sign, cortical lesions and paramagnetic rim lesions. We compared (5 × 2 cross-validation combined *F*-test) the diagnostic performance of 71 machine learning models, each corresponding to a distinct combination of full-count or simplified biomarker inputs, against the baseline dissemination in space McDonald criteria. The aim was to evaluate the multiple sclerosis diagnostic power of combining these biomarkers in an MRI-only diagnostic framework. 51 of the 71 models significantly outperformed the dissemination in space criterion (*P* < 0.05), with balanced accuracy improvements up to 13.0% (confidence interval: [+10.5; +17.0]). The best overall model (random forest, using full-count assessments) achieved 95.7% (confidence interval: [93.2; 99.7]) balanced accuracy; the best simplified model (logistic regression, using only simplified assessments) reached 94.7% with no significant difference with the former (*P* = 0.29). Notably, 12/51 high-performing models used only simplified assessments. To further investigate the models’ generalizability, external validation on two out-of-distribution test sets using bootstrapping (1000 resamples) confirmed these results and highlighted a more robust generalization for the best model using solely simplified biomarkers. On the first external test set (*n* = 37, Verona), the simplified model achieved 97.2% balanced accuracy, while the full-count model reached 93.3% (versus 83.3% for baseline). On the second test set (*n* = 84, prodromal cases), the simplified model achieved 92.6% (versus 60.1% for baseline) showing competitive performance against the full-count model (93.9%). Both models improved all key performance metrics—balanced accuracy, sensitivity, specificity, precision and F1 score—over the baseline on both test sets (all *P* < 0.0001). Within a non-invasive MRI-only diagnostic framework, these results show that the incorporation of advanced imaging biomarkers into the multiple sclerosis-MRI diagnostic criteria significantly enhances the diagnostic accuracy—a statement holding true even when using simplified central vein sign, cortical lesions and paramagnetic rim lesions assessments. The study also provides a publicly available online diagnostic tool, facilitating further interaction, validation and clinical support (https://www.msdiagnostictool.org).

## Teaser

Wynen, Vanden Bulcke *et al*. report that machine learning models incorporating advanced imaging biomarkers—central vein sign, cortical lesions and paramagnetic rim lesions—improve multiple sclerosis -MRI diagnostic accuracy, with improved robustness observed in models using clinically-intuitive simplified biomarker assessment. The authors published an online tool enabling interaction with trained models.

## Graphical Abstract

**Graphical Abstract:**
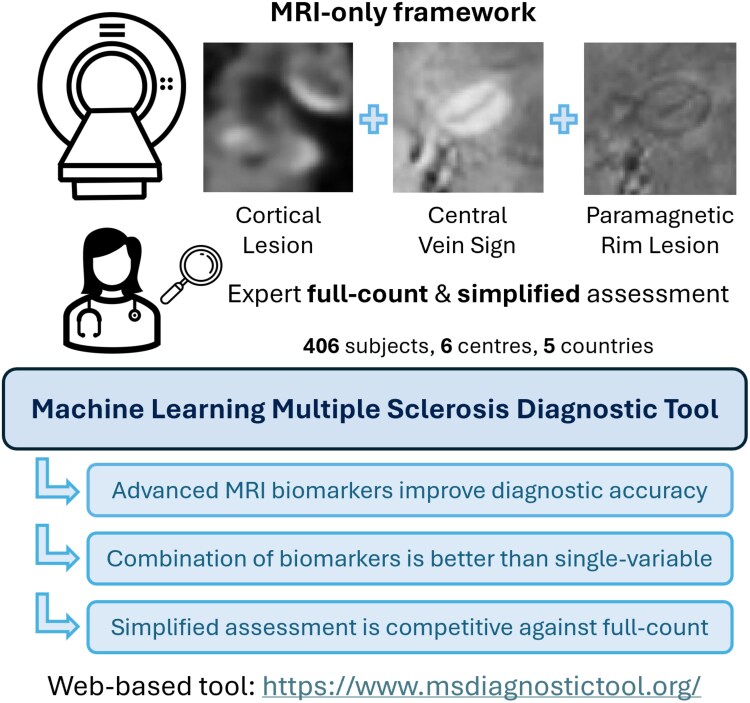

## Introduction

**Figure 1:**
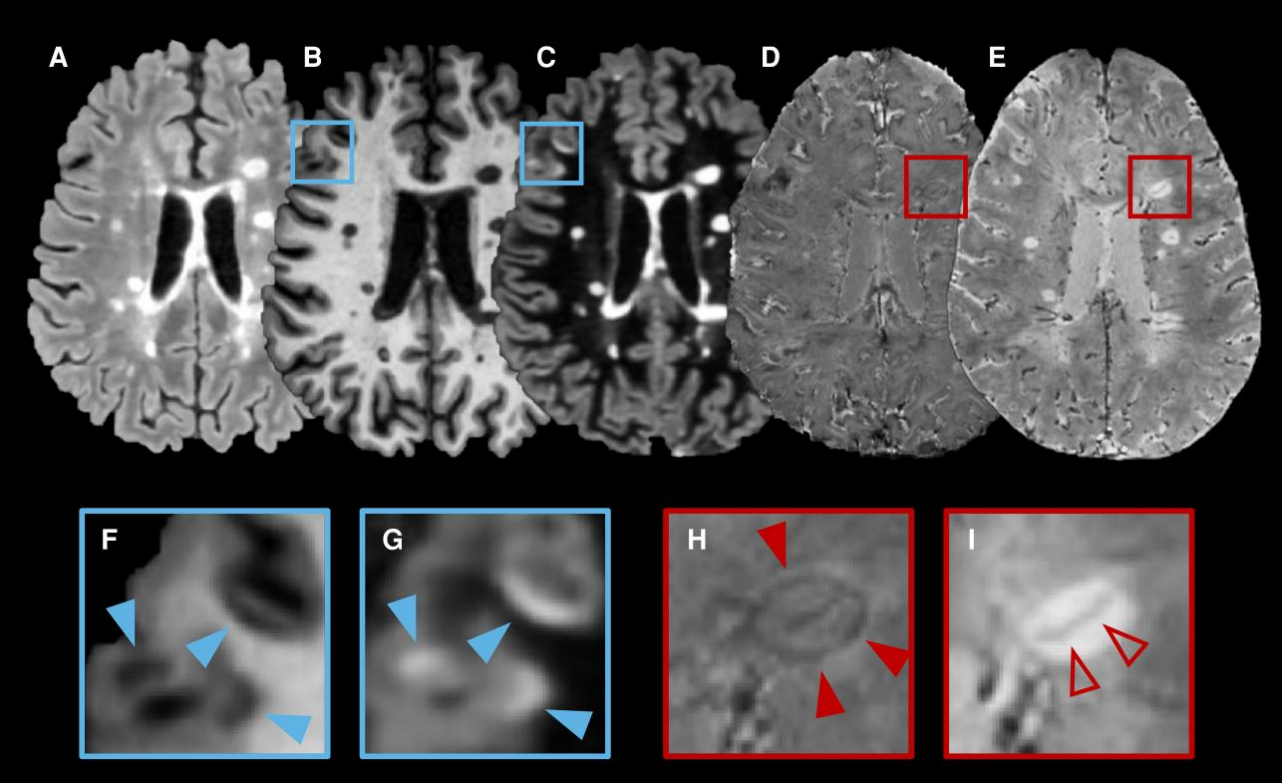
**Advanced magnetic resonance imaging (MRI) biomarkers.** Representative MRI axial view of a 25-year-old man with relapsing-remitting multiple sclerosis (RRMS) showing the three advanced biomarkers on different contrasts: (**A**) Fluid Attenuated Inversion Recovery (FLAIR); (**B**) Magnetization Prepared RApid Gradient Echo (MPRAGE); (**C**) Double Inversion Recovery (DIR); (**D**) Echo Planar Imaging (EPI) unwrapped filtered phase; (**E**) EPI magnitude; (**F–G**): magnified views of cortical lesions (CL) on MPRAGE (**F**) and DIR (**G**) indicated by solid arrows; H: magnified view of a paramagnetic rim lesions (PRL) on EPI unwrapped filtered phase—solid arrows indicating the dark rim; I: magnified view of a lesion showing the central vein sign (CVS) on EPI magnitude—hollow arrow indicating the central vein.

In multiple sclerosis (MS), highly sensitive and specific diagnostic criteria are required to allow timely patient treatment and prevent misdiagnosis. Establishing diagnostic criteria often involves a trade-off between sensitivity and specificity, and the previous 2017 McDonald criteria^1^ tended to favour sensitivity over specificity.^2^ This admittedly led to early detection of the disease^3^ but also increased the risk of incorrectly diagnosing patients with conditions that mimic MS,^4^ potentially overburdening healthcare and harming patients. To address this issue, new advanced MRI biomarkers—the central vein sign (CVS), cortical lesions (CLs) and paramagnetic rim lesions (PRLs) (Fig. 1)—have gained considerable attention in the MS community due to their high specificity for MS, bearing the potential to reduce misdiagnosis while still allowing early detection of the disease.^5,6^ While the CVS reflects the perivenular development of inflammatory demyelination in white matter, PRLs indicate perilesional chronic inflammation, specifically the accumulation of iron-laden microglia/macrophages at the lesion edge after acute inflammation resolves.^7,8^ CLs are focal abnormalities completely within the cortex or spanning both the cortex and the underlying white matter; these were already included in the 2017 revision of the McDonald criteria but remain difficult to detect without specialized MRI techniques.^9,10^ Given the specificity of these new advanced MRI biomarkers for MS, the recently published 2024 revisions to the McDonald criteria propose to incorporate CVS and PRLs as additional MRI features (CLs being formally already included in the 2017 revision) to enhance diagnostic accuracy.^11^ However, the full-count (exhaustively detecting all occurrences) assessment of these advanced biomarkers is time-consuming and often incompatible with clinical practice. In this context, the effects of using more practical, simplified biomarker assessments (classifying each subject as positive or negative for a given biomarker), as well as the impact on MS diagnostic performance of combining these advanced MRI biomarkers remains uncertain.

In line with broader trends demonstrating the potential of machine learning (ML) for diagnosing neurological diseases,^12,13^ this study investigates ML as a means to (i) improve MS diagnostic accuracy, and (ii) facilitate the incorporation of CVS, CLs and PRLs—with or without the existing dissemination in space (DIS) imaging criteria –^1,14^ into an exclusively MRI-based diagnostic framework, thus independently of any other clinical or paraclinical feature. In particular, we analyse ML models trained with a unique combination of input covariates and compare their performance against previously accepted DIS criteria.^1^ We expect that the inclusion and combination of MS-specific MRI biomarkers will improve specificity while maintaining the high sensitivity of the current DIS criteria. Finally, this paper comes along with a publicly available online tool for MS diagnosis, allowing users to explore and interact with the ML models discussed hereafter (https://www.msdiagnostictool.org).

## Materials and methods

### Dataset and covariates

**Figure 2:**
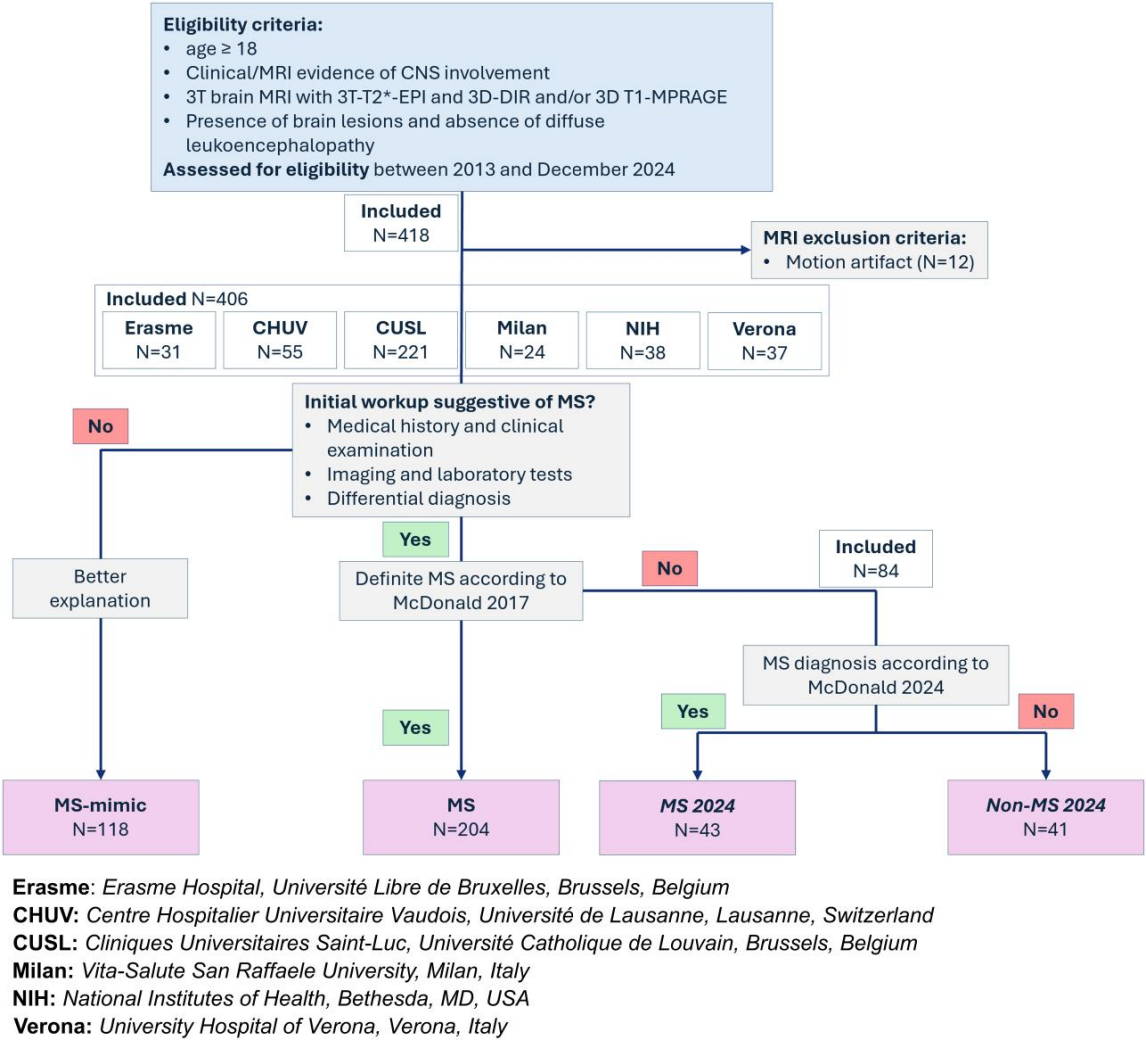
**Participant flow chart. Summarizes patients’ progress through the study.** CHUV, Centre Hospitalier Universitaire Vaudois; CNS, central nervous system; CUSL, Cliniques Universitaires Saint-Luc; EPI, echo planar imaging; DIR, double inversion recovery; MPRAGE, magnetization prepared—rapid gradient echo; MRI, magnetic resonance imaging; MS, multiple sclerosis; NIH, National Institutes of Health.

This study uses a retrospective convenience dataset of 406 patients from six centres across four countries: Erasme (Erasme Hospital, Université Libre de Bruxelles, Brussels, Belgium), CHUV (Centre Hospitalier Universitaire Vaudois, Université de Lausanne, Lausanne, Switzerland), CUSL (Cliniques Universitaires Saint-Luc, Université Catholique de Louvain, Brussels, Belgium), Milan (Vita-Salute San Raffaele University, Milan, Italy), NIH (National Institutes of Health, Bethesda, MD, USA) and Verona (University Hospital of Verona, Verona, Italy). In adult patients with clinical/MRI evidence of central nervous system (CNS) involvement, individuals were first classified as having an MS diagnosis according to McDonald 2017,^1^ or having MS-mimic conditions according to internationally published diagnostic criteria^1^ (MS-mimic/MS, 118/204). The MS-mimic group included other inflammatory neurological disorders (OIND), non-inflammatory neurological disorders (NIND) and neurotropic viral infections (NV) (see Table 1 for diagnostic categories and Suppl. Sec. 1.3 for detailed pathologies). Cases having a suspected prodromal MS pathology—defined as patients not meeting the 2017 McDonald criteria and primarily composed of those with clinically or radiologically isolated syndrome (resp. CIS or RIS)—were instead classified as MS/Non-MS (43/41) according to the 2024 revision of the McDonald criteria.^11^ Patient eligibility criteria included (i) age ≥18 years, (ii) clinical/MRI evidence of CNS involvement, (iii) presence of brain lesions and absence of diffuse leukoencephalopathy (without any focal lesion), and (iv) availability of 3T 3-dimensional (3D) segmented T2*-weighted echo planar imaging (EPI) for CVS and PRL assessment, and double inversion recovery (DIR) and/or T1-weighted magnetization prepared rapid gradient echo images (MPRAGE) for CL assessment (refer to Supplementary Table 1 for a comprehensive overview of acquisition parameters). Figure 2 shows a flow chart of participants inclusion and Table 1 reports baseline demographic and clinical characteristics. Only fully anonymized imaging (in the brain imaging data structure—BIDS,^15,16^ or tabular data) and clinical data, with all personal identifiers removed, were shared between participating centres under institutional data transfer agreements compliant with the General Data Protection Regulation (GDPR). Study procedures received approval from an ethical standards committee on human experimentation (Brussels Saint-Luc IRB B4032020000104), and written informed consent according to the declaration of Helsinki was obtained from all participants prior to participation.

**Table 1:** Demographic and clinical characteristics of patients belonging to seven different datasets separated in a training set and two independent test sets

	Training Set						Test Sets	
Clinical Site	Erasme	CHUV	CUSL	Milan	NIH	Total	Verona	CUSL prodromal
Participants, *n*	31	55	137	24	38	285	37	84
Women, *n* (%)	18 (58)	36 (65)	90 (66)	20 (83)	18 (47)	182 (64)	27 (73)	66 (79)
Age, mean (range)	49 (29–69)	47 (24–76)	45 (22–77)	43 (20–60)	53 (21–75)	47 (20, 77)	44 (23–67)	42 (18–69)
MS, *n* (RRMS, SPMS, PPMS)	30 (7, 17, 6)	46 (29, 9, 8)	99 (68, 21, 10)	10 (9, 0, 1)	0 (0, 0, 0)	185 (113, 47, 25)	19 (18, 1, 0)	43^a^
Non-MS, *n* (OIND, NIND, NV)	1 (1, 0, 0)	9 (7, 2, 0)	38 (13, 25, 0)	14 (8, 6, 0)	38 (9, 10, 19)	100 (38, 43, 19)	18 (15, 3, 0)	41^a^

The datasets originate from six different centres; Erasme, Erasme Hospital, Université Libre de Bruxelles, Brussels, Belgium; CHUV, Centre Hospitalier Universitaire Vaudois, Université de Lausanne, Lausanne, Switzerland; CUSL, Cliniques Universitaires Saint-Luc, Université Catholique de Louvain, Brussels, Belgium; Milan, Vita-Salute San Raffaele University, Milan, Italy; NIH, National Institutes of Health, Bethesda, MD, USA; Verona, University Hospital of Verona, Verona, Italy. Patients with evidence of CNS involvement were classified as having a definite MS diagnosis according to McDonald 2017,^1^ or having MS-mimicking conditions, composed of OIND, NIND, and NV (detailed die, according to internationally published diagnostic criteria.

^a^The prodromal test set is composed of prodromal MS cases (per 2017 McDonald criteria) that were stratified as MS/non-MS per the 2024 revision of McDonald criteria^11^ (for this particular cohort, unfulfilment of 2024 McDonald criteria result in a non-MS diagnosis). Central nervous system, CNS, MS, multiple sclerosis; RRMS, remitting-relapsing MS; SPMS, secondary progressive MS; PPMS, primary progressive MS; OIND, other inflammatory/infectious neurologic diseases; NIND, noninflammatory neurologic diseases; NV, neurotropic viruses.

Covariates used in this study included demographic information (*age* and *sex assigned at birth*), and MRI covariates grouped into four categories: one DIS criterion—according to McDonald 2017 (*DIS*)—and three advanced MRI biomarkers—CVS, CL and PRL (Fig. 1). More specifically, DIS was defined as the presence of at least one or more T2 hyperintense lesion in at least two of the typical areas (peri-ventricular, (juxta-)cortical, infra-tentorial, spinal-cord).^1^ In addition, all occurrences of CL and PRL were exhaustively counted (full-count), and participants were also dichotomized as having the presence of at least one CL and/or one PRL for simplified assessment. For the CVS, the percentage of perivenular lesions (*%CVS*) was determined across all eligible brain lesions^17^ in each participant (full-count). Additionally, each participant was dichotomized as CVS-positive/CVS-negative/not-applicable based on the ‘Select-3*’^14^ and ‘Select-6*’^18^ simplified algorithms, where a scan was considered as CVS-positive if there were respectively ≥3 or ≥6 eligible lesions that met the CVS-positive NAIMS criteria;^17^ not applicable if there were <3 eligible lesions. See the supplementary materials for details.

In each patient, CVS, CL and PRL analysis was independently assessed following established guidelines and methods by two trained investigators (P.M. and S.B. for Erasme, CUSL, CHUV and Verona cases; M.A. and M.S.M. for Milan and NIH cases), each unaware of the other's analysis and blinded to participants’ diagnosis. All non-MS cases that were CVS-positive or bearing ≥1 PRL or ≥1 CL were jointly reviewed by M.A. and P.M. for final adjudication. The intraclass correlation coefficient (ICC) inter-rater agreement, computed on the training set, was 0.995 (95% confidence interval [CI] 0.980–0.999, *P* < 0.001) for CVS assessment; 0.998 (95% CI 0.995–0.999, *P* < 0.001) for CL assessment; and 0.986 (95% CI 0.950–0.996, *P* < 0.001) for PRL assessment. Below is a summary of the covariates:

- Age
- Sex assigned at birth
- DIS according to McDonald 2017^1^
- Full-count assessments: total percentage of CVS positive lesions (%CVS), total CL and PRL counts (resp. #CL and #PRL)
- Simplified assessments: CVS Select-3*^14^ and Select-6*^18^ algorithms, having ≥1 CL/PRL (resp. CL1/PRL1)

### Machine learning framework and evaluation protocol

This section outlines the approach employed to investigate the application of ML in diagnosing MS using only cross-sectional demographic and radiological data. The diagnostic task was formulated as a binary classification problem, using covariates detailed in the previous subsection, to predict a binary output designating MS presence (1) or absence (0). Following statistical analysis (cf. infra), feature selection excluded *Age* and *Sex assigned at birth* due to the insufficient discriminatory power of these variables. The ML algorithms selected for this study include logistic regression^19^ (LR), decision tree,^20^ random forest (RF),^21^ K-nearest neighbours classifier (KNN),^22^ support vector classifier (SVC),^23^ and extreme gradient boosting (XGB).^24^ These algorithms were chosen due to their widespread use, demonstrated performance and accessibility via Python libraries. Finally, *balanced accuracy—*defined as the average between specificity and sensitivity*—*was selected as the performance metric to be maximized. This metric was chosen to ensure an optimal trade-off between sensitivity and specificity, thereby providing a robust evaluation of model performance. Prior to conducting any analyses, the dataset was partitioned into a training set, comprising data from five centres (*n* & 285), and two independent test sets (*n* & 121), selected specifically to evaluate the generalizability of the ML models to unseen and heterogeneous data (see Table 1). The first test set included data from Verona, Italy (*n* = 37) to ensure scanner and geographical diversity, while the second included prodromal cases from CUSL (*n* = 84) to test the models against the upcoming 2024 McDonald criteria.^11^

Using the training dataset, step 1 of the analysis identified the optimal algorithm-combination pairs by selecting the ML algorithm that maximized performance for each of the 71 possible covariate combinations (e.g. *%CVS+#PRL* or Select-3*+CL1  *+*  *PRL1*; refer to Suppl. Sec. 1.3. for more detailed explanations). More specifically, using 10-fold cross-validation (CV), the algorithm achieving the highest balanced accuracy was chosen for each combination. The optimal classification cutoff was determined by averaging each folds’ optimal cutoff, maximizing balanced accuracy. Steps 2–5 were also performed on the training dataset (Suppl. Sec. 1.3.4.): Step 2 evaluates all algorithm-combination pairs against the DIS (hereafter called ‘baseline’), categorizing them as either significantly better or not, and ranking them by balanced accuracy improvement relative to the baseline. The best overall pair (*M_Best_*) and the best pair using only simplified variables (*M_Simplified_*) were identified. Step 3 tested whether *M_Best_* was significantly superior to other pairs, while Step 4 did the same for *M_Simplified_* within simplified-variable models. Step 5 directly compared *M_Best_* and *M_Simplified_* against each other.

To ensure model robustness and generalizability, performance was evaluated on two unseen datasets, mitigating the risk of overfitting. All models, but specifically *M_Best_* and *M_Simplified_*, were retrained on the full training set, to maximize available information. All models’ performance was then assessed on the two independent test sets and compared to the baseline DIS performance by conducting a holistic evaluation using balanced accuracy, sensitivity, specificity, precision and F1 score metrics. Finally, the performance of *M_Best_* and *M_Simplified_* was further compared to that of all single-variable models on the prodromal test dataset (Test set 2) to evaluate the added value of combining multiple biomarkers in the context of the new 2024 diagnostic criteria.

### Online diagnostic tool

**Figure 3:**
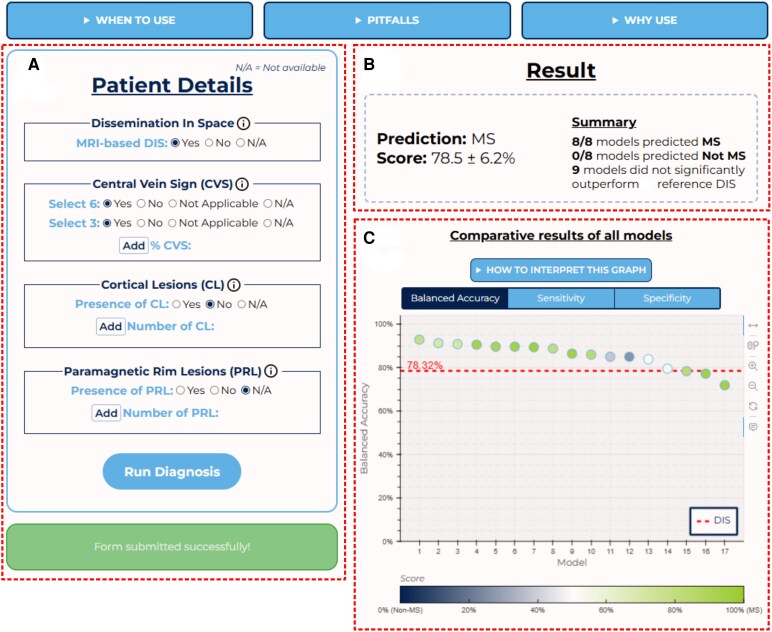
**Screenshots of the proposed online tool, available https://www.msdiagnostictool.org.** On the left (**A**) lies the form enabling the user to enter relevant patient-related data and run multiple machine learning models. On the right, the website returns the results of the inquired models with different outputs: (**B**) the prediction of the best performing model—determined by performance across both test sets—alongside a summary of all models, grouped by whether or not they significantly outperformed the baseline (McDonald dissemination in space (DIS)) in training; (**C**) a graphical chart displaying the prediction of all models, sorted by their performance on the two test sets. DIS, dissemination in space; MS, multiple sclerosis.

Accompanying this paper, the authors have made available an open-access online tool (see Fig. 3, https://www.msdiagnostictool.org) following an MDCalc^25^ fashion, which enables users to input patient data and instantly receive diagnostic predictions from the study's models, facilitating direct application of the research findings and reproducibility of the results. To ensure the reliability and interpretability of the models deployed on the website, several measures were implemented, including model calibration, uncertainty quantification, user-friendly output presentation and SHAP (SHapley Additive exPlanations)-based explainability.^26^

Since the raw outputs of classifiers often do not represent true posterior probabilities,^27^ model calibration is essential to adjust predicted probabilities to better reflect actual likelihoods, enabling reliable interpretation and comparison of different model outputs. We used Platt’s calibration method^28^ from scikit-learn with 5-fold CV, which performs well on small datasets,^27^ thus producing five distinct calibrated models for each algorithm-combination pair. The website displays as a final output score the average prediction and associated standard deviation across the 5-fold recalibrated models, providing both a robust estimate and a measure of uncertainty of the algorithm-combination pair.

To mitigate potential confusion from displaying the outputs of up to 71 models, we implemented three summarized visual aids (Fig. 3). The first (Fig. 3A) shows the best-performing model—determined by performance across both test sets—alongside a summary of all models, grouped by whether or not they significantly outperformed DIS in training. Additionally, a summary chart (Fig. 3B) displays the outputs of all models, sorted by their performance on the two test sets, while a summary table provides detailed information about each model, including its algorithm, input combination and key performance metrics. This presentation allows users to compare predictions across models and evaluate their consistency.

To enhance explainability, we employed SHAP values,^26^ which decompose predictions into feature contributions, enabling users to understand how specific variables influence the output. By ensuring transparency and enabling users to understand the models’ decision-making process, the four measures we developed in this section support a practical application of the proposed tool in clinical settings.

### Statistical analysis

The statistical analysis involved ANOVA *F*-value feature selection, excluding age and sex due to insufficient discriminatory power. Missing data handling was not required, as the strict inclusion criteria described above ensured that the final cohort contained no missing values. Step 1 used a 10-fold CV to select the optimal algorithm for each combination of features. Steps 2–5 used a 5 × 2CV combined *F*-test,^29,30^ a robust statistical method for comparing classifier performance. This test repeats a two-fold CV five times to estimate performance variability and computes an *F*-statistic to determine whether the observed differences between models are statistically significant. A supplementary Benjamini–Hochberg correction was applied to correct *P*-values for multiple comparison. To assess the models’ generalizability, bootstrapping with 1000 resamples was performed to construct confidence intervals for all metrics and to perform pairwise one-sided Wilcoxon signed-rank tests aiming to determine whether *M_Best_* and *M_Simplified_* significantly outperformed the baseline across these metrics. Finally, the same Wilcoxon test was used to assess whether *M_Best_* and *M_Simplified_* showed significantly superior performance to each single-variable model with the new McDonald 2024 criteria^11^ as a reference. The significance level was set to 0.05 for all tests.

## Results

Among the 71 algorithm-combination pairs evaluated, step 2 identified 51 models demonstrating significant (*P* < 0.05) superior performance compared to DIS, with balanced accuracy ranging from 88.1% (95% CI: [85.4; +91.6]) to 95.7% (95% CI: [93.2; 99.7]) compared to 82.8% for DIS (Supplementary Table 2). Only two models lost significance after *P*-value correction for multiple comparisons. Notably, 12 of the 51 models used only simplified assessments. Frequency analysis revealed that CVS, PRL, CL and DIS were included in 49, 38, 35 and 26 of these models, respectively, with 24 models combining CVS, PRL and CL, and 14 using two or fewer input variables. Furthermore, 90.7%, 79.2%, 72.9%, and 72.2% of algorithm-combination pairs, including CVS, PRL, CL and DIS, respectively, significantly outperformed DIS alone. More particularly, among the models with a single-variable input, the RF classifier using *%CVS* and the LR using Select-3* were the only ones that showed significantly superior performance to DIS.

**Figure 4:**
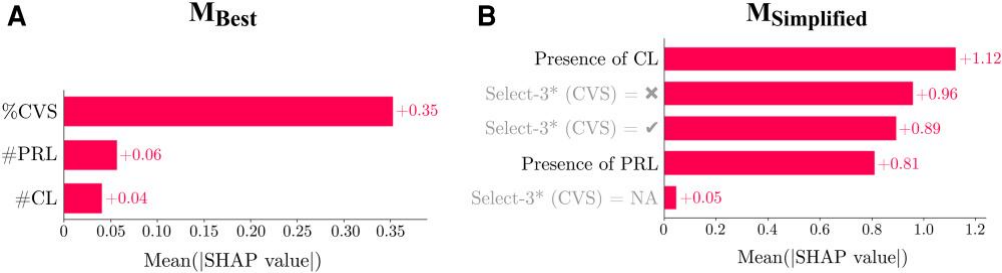
**SHapley additive exPlanations (SHAP)-derived summary plots of the best model (*M_Best_*—A) and the best model using only simplified assessment (*M_Simplified_*—B).**  *M_Best_* is a random forest classifier using *%CVS*, *#CL* and *#PRL* while *M_Simplified_* is a logistic regression model using Select-3*, *CL1* and *PRL1* as input (cf. section ‘Dataset and Covariates’ for detailed explanation of covariates). The summary plots show the importance of each feature on the model’s output prediction. CVS, central vein sign; CL, cortical lesion; PRL, paramagnetic rim lesion .

*M_Best_* was identified as an RF classifier using *%CVS*, *#CL* and *#PRL* and *M_Simplified_* as a LR model using Select-3*, *CL1* and *PRL1*. SHAP-based interpretability plots for these models (Fig. 4) provide insights into the contribution of individual biomarkers to the predictions (Section ‘Online diagnostic tool’). This analysis highlighted CVS as the most influential variable in both models (77.7% for *M_Best_* and 49.6% for *M_Simplified_*), aligning with previous findings.^5^ Interestingly, and again in line with our previous observation,^5^ CL and PRL contributed more to the output in *M_Simplified_* compared to *M_Best_* (29.2% and 21.1% versus 13.3% and 8.8%, respectively). Step 3 found no statistically significant difference in balanced accuracy among the top 36 models outperforming DIS (including a RF classifier with the single *%CVS* covariate as input), nor did step 4 among all models using only simplified assessments and outperforming DIS. Moreover, *M_Best_* and *M_Simplified_* showed no statistically significant difference in performance (*P* & 0.29).

The generalization study results, summarized in Table 2, reveal that both *M_Best_* and *M_Simplified_* improved all metrics (balanced accuracy, specificity, sensitivity, precision and F1) compared to DIS in both test sets (*P* < 0.0001). Specifically, on the first test set (Verona), *M_Best_* and *M_Simplified_* achieved balanced accuracies of 93.3% and 97.2%, respectively, compared to 83.3% for DIS. On the second test set (CUSL Prodromal), they achieved 93.9% and 92.6%, respectively, compared to 60.1% for DIS. Notably, *M_Simplified_* demonstrated the highest performance across all metrics on Test set 1, while *M_Best_* achieved the highest scores on Test set 2. As detailed in Supplementary Table 3, both *M_Best_* and *M_Simplified_* also significantly outperformed all single-variable models on Test set 2, confirming the added value of combining multiple expert-assessed biomarkers.

**Table 2:** Performance of the McDonald dissemination in space (DIS), the best model, and the best model using only simplified assessment on the two independent test sets; a test set (test set 1) of 37 MS/MS-mimic (19/18) patients and a second test set (test set 2) of prodromal MS patients (84) with clinically or radiologically isolated syndromes stratified as MS/Non-MS (43/41) by applying the 2024 revision of McDonald criteria^11^

Model		(Baseline)	*M_Best_*	*M_Simplified_*
Input variables		*DIS*	*%CVS, #PRL, #CL*	Select-3*, CL1, PRL1
Balanced accuracy (%) [CI]	Test set 1	83.3 [71.9; 94.1]	93.3 [83.3; 100]	**97.2 [90.6; 100]**
	Test set 2	60.1 [49.9; 69.3]	**93.9 [88.1; 98.8]**	92.6 [86.3; 97.6]
Sensitivity (%) [CI]	Test set 1	**100 [100; 100]**	**100 [100;100]**	**100 [100; 100]**
	Test set 2	86.0 [73.7; 95.1]	**95.2 [88.4; 100]**	**95.2 [87.8; 100]**
Specificity (%) [CI]	Test set 1	66.7 [43.8; 88.2]	86.7 [66.7; 100]	**94.4 [81.2; 100]**
	Test set 2	34.1 [18.2; 48.6]	**92.5 [83.8; 100]**	90.0 [80.0; 97.7]
Precision (%) [CI]	Test set 1	75.0 [57.1; 91.7]	89.5 [73.7; 100]	**94.7 [81.8; 100]**
	Test set 2	57.8 [44.6; 68.7]	**93.0 [84.6; 100]**	90.9 [81.8; 97.9]
F1 Score (%) [CI]	Test set 1	85.7 [72.7; 95.6]	94.4 [84.8; 100]	**97.3 [90; 100]**
	Test set 2	69.2 [57.4; 78.0]	**94.1 [88.4; 98.8]**	93.0 [87.0; 97.9]

CI, confidence interval; CVS, confidence interval; CL, confidence interval; PRL, paramagnetic rim lesions. Values highlighted in bold represent the highest performance metric score across all three models on a specific test set.

## Discussion

**Figure 5:**
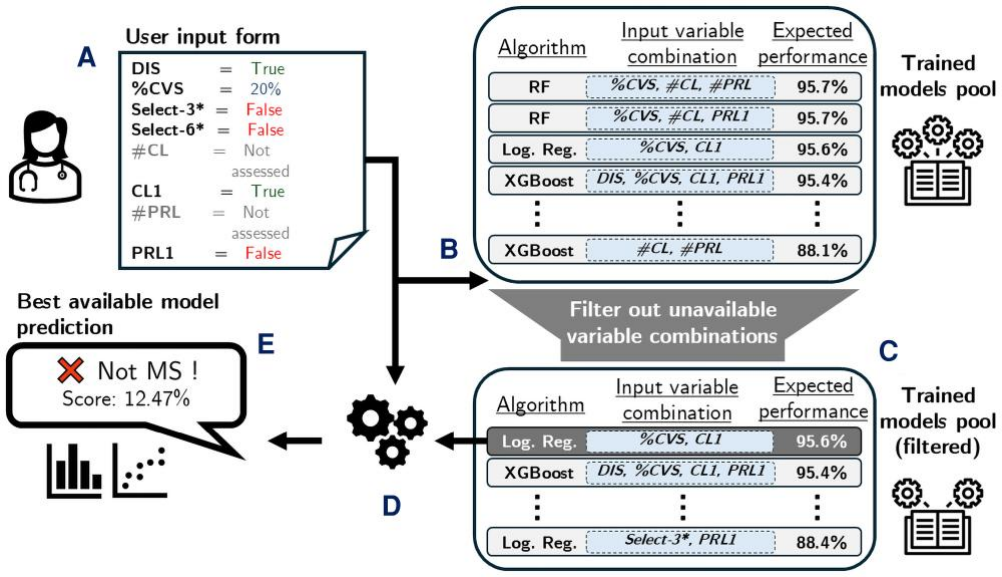
**Schematic outline and illustrative example of the proposed online tool.** (**A)** Once on the website, the user fills in a form with the patient’s available information (biomarker and dissemination in space (DIS) assessments). (B) The trained models pool, where each model is associated with a specific combination of input variables and an expected performance (cf. Sec. ‘Dataset and covariates’), is then filtered to retain only those models where all required input variables are provided by the user. (C–D) Next, the model yielding highest expected performance is selected (**C**) to be inferred on the provided user input (**D**). (E) Finally, the best available model’s prediction is sent back to the user with an associated score and interpretability visuals. Of note, all models’ predictions and interpretability visuals are also accessible upon user request. Performance is characterized by balanced accuracy. CVS, central vein sign; CL, cortical lesion; logistic regr., logistic regression; MS, multiple sclerosis; PRL, paramagnetic rim lesion.Select-3/6*: refer to Sec. ‘Dataset and covariates’.

This multicentre study aims to integrate advanced MRI biomarkers into an exclusively MRI-based diagnostic approach through ML. Specifically, we use a dataset of 204 MS, 118 MS-mimic and 84 prodromal MS cases from six centres across five countries to assess ML’s ability to determine the diagnostic value of different combinations of the CVS, PRL and CL with or without existing MRI DIS diagnostic criteria. Additionally, we examine the competitiveness of more clinically intuitive simplified assessments of the CVS, PRL and CL against their full-count counterparts and introduce a publicly accessible online tool for interacting with the trained models (https://www.msdiagnostictool.org, Fig. 5).

In line with recent research,^5,6^ our results indicate that combining advanced MRI biomarkers—especially when using simplified assessments—significantly enhances diagnostic accuracy. Notably, both *M_Best_* and *M_Simplified_* (cf. 2.2) used all three biomarkers (CVS, PRL and CL) without DIS, and achieved average balanced accuracies on the training set of 95.7% and 94.7% respectively, representing an improvement of 13% (95% CI [+10.5; +17]) and +11.4% (95% CI [+7.7; +13.3]) over established DIS criteria. Based on our frequency analysis and SHAP-derived interpretability plots, CVS emerges as the most diagnostically powerful biomarker, with PRL and CL close behind. Combining CVS with PRL and/or CL further improved differential diagnosis. Interestingly, no significant performance difference was observed between *M_Best_* and *M_Simplified_*. This finding supports the notion that simplified diagnostic algorithms carry sufficient discriminative power, reinforcing the rationale behind their integration in the newly revised McDonald criteria, ensuring both wide applicability in clinical practice and high diagnostic accuracy. Model selection and tuning were conducted using 10-fold cross-validation for each of the input variable combinations, and comparisons with baseline DIS and between models used the 5 × 2CV combined *F*-test. To test generalizability, we evaluated *M_Best_*, and *M_Simplified_* on two independent, out-of-domain, test sets: a first test set from an independent centre with definite diagnoses according to the 2017 McDonald criteria (as it was the case for the diagnoses included in the training set), and a second test set from a centre with prodromal cases dichotomized as MS/non-MS according to the 2024 McDonald criteria (diagnostic scenario unseen in the training set). Both models showed outstanding performance on the test sets, considerably improving the specificity over baseline DIS while maintaining high sensitivity. Specifically, both models achieved excellent specificity (92.5% and 90.0%) on the second test set, while DIS’s specificity dropped to 34.1%. In line with these findings, additional analyses on the prodromal dataset (Table S3) confirmed that both *M_Best_*, and *M_Simplified_* significantly outperformed all single-variable models, including those based solely on CVS, further emphasizing the complementary value of combining multiple biomarkers. Additionally, *M_Simplified_* showed a performance close to that of *M_Best_* on the second test set, and even outperformed it on the first one (Table 2). These results highlight the practicality of these time-saving criteria when combined. Furthermore, results on the second test set, composed of prodromal MS cases, show the ability of a non-invasive MRI cross-sectional evaluation to accurately diagnose MS according to the new 2024 revised McDonald criteria.^11^ This suggests that an exclusively imaging-based approach may often represent an alternative to more invasive procedures (e.g. lumbar puncture), in centres where an advanced MRI protocol is available. Overall, this study showed that ML models can uncover complex biomarker interactions, yielding more accurate diagnoses than traditional biomarker decision-tree (or ‘if-else’) like approaches, albeit featuring a slight diminishment in interpretability and applicability.

All 71 ML models implemented in this study are available on a dedicated website to enable researchers and clinicians to interact with them (Fig. 5). Indeed, this tool is intended as a consultative aid for clinicians, facilitating the interpretation of a combined advanced MRI biomarkers work-up, designed to complement and enhance clinical decision-making rather than replace established diagnostic criteria. After an inquiry, a user can consult the outputs of each model that was used. Notably, a series of measures were taken to improve the interpretability and explainability of the models (cf. Section ‘Online diagnostic tool’), thereby mitigating the diminishment in applicability mentioned above. Moreover, because no input data are stored and only biomarker assessment is needed, the website fully respects patient data privacy. However, at this stage, the tool should be regarded as a research instrument, developed to facilitate reproducibility, external validation and exploration of advanced MRI biomarkers within a standardized framework. It is not yet an approved diagnostic device and should therefore not be used for standalone clinical decision-making. In the future, upon prospective validation, this approach could be integrated into existing diagnostic workflows as a supportive aid, potentially reducing reliance on more invasive procedures.

A limitation of our study is that dissemination in time (DIT) and CSF-restricted oligoclonal bands were not considered in the analysis, although they were included in the ground truth diagnosis. This was primarily due to the non-invasive, cross-sectional MRI design of our study. Notably, the 2024 revision of the McDonald criteria^11^ reduces the emphasis on DIT and lumbar puncture and favours the assessment of MS-specific advanced MRI biomarkers (using the simplified methods Select-6* and PRL1) to enhance specificity and facilitate clinical implementation.^14,18,31^ Although the current limited clinical availability of the optimized MRI sequences required for CVS, PRL and CL detection hinders the immediate widespread adoption of our tool, the recent inclusion of these biomarkers into the newly updated MS diagnostic criteria represent a strong incentive to incorporate such sequences into standard clinical MRI protocols moving forward. The real-world performance of our framework could also be influenced by variability in imaging protocols and the potential for inter-observer differences in identifying CVS, CL and PRL. The use of simplified assessments and the growing number of proposed methods aiming to automate their assessment (e.g.^32-36^) are key in this regard, as they would help mitigate variability and enhance clinical feasibility. In our study, however, the final model inputs were the expert-assessed MRI biomarker evaluations rather than raw image data, which likely reduced the impact of inter-scanner variability on model performance. The relatively small number of patients included represents another limitation of this study, primarily due to the inclusion criteria requiring a specific advanced MRI protocol (for advanced MRI biomarker assessment, cf. Suppl. Sec. 1.1.). While it minimizes bias from biomarker assessment across different sequences and field strengths, this convenience sample may not fully represent the demographic or MRI protocol diversity found in clinical practice. However, a dataset with such a homogeneous MRI protocol from six different centres is unprecedented in MS research. Additionally, the dataset used in this work also include a high number of MS-mimic inflammatory cases, especially in the test set. These cases are rarer and challenging to collect and have likely reduced the statistical power of our results, as inflammatory MS-mimics are typically harder to distinguish from MS compared to other MS-mimic disorders. Thus, our dataset composition should not affect the main conclusion of this study. Future research should focus on increasing the sample size and exploring rule-based ML to provide experts with pragmatic and directly interpretable computer-aided diagnosis. In combination with recent and emerging automated segmentation methods for CVS, PRL and CL detection,^32^ the proposed framework could naturally evolve into a fully automated diagnostic pipeline, enabling rapid, scalable and reproducible assessment. Beyond MRI, optical coherence tomography (OCT) might also represent a promising complementary non-invasive technique to refine diagnostic accuracy and may be considered in future multimodal frameworks.^37^

In conclusion, this study explores through ML the incorporation of advanced MRI biomarkers combinations into an exclusively MRI-based framework for the diagnosis of MS. Our findings indicate that ML models combining CVS, CL *and* PRL show the highest MS diagnostic performance and clearly outperform the DIS MRI criterion for MS diagnosis. Notably, this holds true even when using exclusively simplified MRI biomarkers assessments, which demonstrated similar generalization power to out-of-domain test sets compared to their full-count counterparts. This approach has the potential to improve MS diagnosis, while reducing the need for supplementary invasive exams without compromising clinical practicality. The study also provides a publicly available online tool to enable further interaction with the trained ML models, facilitating further validation (https://www.msdiagnostictool.org).

## Supplementary Material

fcag079_Supplementary_Data

## Data Availability

Qualified researchers can access the individual patient data of this study upon reasonable request and material transfer agreement between institutes. The trained models of the study are available at https://www.msdiagnostictool.org/. Source code used in the paper is available at https://github.com/maxencewynen/MS-Diagnostic-Tool-UCLouvain.
