# Supplementary material for: Machine learning-based combination of the central vein sign, cortical lesions and paramagnetic rim lesions: a web-based tool for the diagnosis of multiple sclerosis
Source: Brain Commun. 2026 Mar 11;8(2):fcag079. doi: 10.1093/braincomms/fcag079 (PMC13010066; doi:10.1093/braincomms/fcag079)
Supplement: fcag079_Supplementary_Data [file fcag079_supplementary_data.docx]

### **Supplementary materials**

1. Supplementary methods

**1.1. MRI acquisition**

MRI studies were performed on 8 different 3T MRI scanners: 4 Philips Intera, Ingenia or Achieva scanners (Philips Medical Systems, The Netherlands), 3 Siemens Skyra or Prisma scanners (Siemens AG, Germany), and 1 General Electric Signa Premier (GE Healthcare, Waukesha, Wisconsin, USA). In all centers, 3D T2-Fluid Attenuated Inversion Recovery (T2-FLAIR) images and submillimeter isotropic 3D segmented T2*-weighted EPI,^1–3^ providing both magnitude and phase images, were acquired during, or immediately after intravenous injection of a single dose (0.1mmol/kg) of gadolinium-based contrast material for the detection of CVS and PRL imaging biomarkers. The same 3D T2*-EPI sequence^1–3^ was adapted and optimized to the different MRI scanners with minimal parameter modification (Table S1). CL assessment was performed on 3D-DIR and 3D-MPRAGE images from Brussels, while on synthetic-DIR (generated from T2/T1 images,^4^ see below) and 3D-MPRAGE at the other centers.^5,6^ Additional routine MRI scans were acquired for clinical use, including post-contrast T1-weighted sequences.

**Supplementary Table 1**: Whole-brain 3D segmented (multi-shot) echo-planar (EPI) sequence parameters for all the scanners implemented in the study. Erasme (Erasme Hospital, Université Libre de Bruxelles, Brussels, Belgium), CHUV (Centre Hospitalier Universitaire Vaudois, Université de Lausanne, Lausanne, Switzerland), CUSL (Cliniques Universitaires Saint-Luc, Université Catholique de Louvain, Brussels, Belgium), Milan (Vita-Salute San Raffaele University, Milan, Italy), NIH (National Institutes of Health, Bethesda, MD, USA), Verona (University Hospital of Verona, Verona, Italy).

| **Scanner** | Siemens Skyra | Siemens  Skyra/Prisma | GE Premier | Philips Intera | Philips Ingenia | Philips Ingenia/Achieva |
| --- | --- | --- | --- | --- | --- | --- |
| Field Strength | 3T | 3T | 3T | 3T | 3T | 3T |
| Site | NIH | CHUV | CUSL | NIH/Milan | Erasme | Verona |
| Receive channels | 32 | 64 | 48 | 8 | 16 | ? |
| Imaging plane | Sagittal | Sagittal | Sagittal | Sagittal | Sagittal | Sagittal |
| Imaging resolution in-plane (mm) | 0.65 | 0.65 | 0.67 | 0.55 | 0.55 | 0.67/0.55 |
| Slice thickness (mm) | 0.65 | 0.65 | 0.67 | 0.55 | 0.55 | 0.67/0.55 |
| # slices | 256 | 288 | 355 | 336 | 336 | 284/336 |
| Repetition time (TR, ms) | 64 | 64 | 80.2 | 53 | 63.8 | 67/54 |
| Echo time  (TE, ms) | 35 | 35 | 35 | 29 | 29.4 | 35/29 |
| Flip angle (deg) | 10° | 10° | 18° | 10° | 10° | 10° |
| Acquisition Time (min:sec) | 5:46 | 6:20 | 4:18 | 4:40 | 4:40 | 6:33/5:27 |

**1.2. MS-mimics conditions**

The MS-mimic cohort included individuals with other inflammatory neurologic diseases (OIND), neurotropic viral infections (NV), or noninflammatory neurologic diseases (NIND), classified according to established diagnostic criteria (see main text-). More specifically in the training set:

- OIND category comprises 12 cases of neuromyelitis optica spectrum disorder (NMOSD; 11 AQP4 antibody–positive or 1 seronegative); 3 cases of myelin oligodendrocyte glycoprotein antibody–associated disease (MOGAD); 6 cases of systemic lupus erythematosus (SLE), 5 cases of Susac syndrome, 6 cases of Behçet disease, 3 cases of Sjögren disease, 2 cases of sarcoidosis, and 1 case of ANCA-associated vasculitis.
- NV category comprises 9 cases with human T-lymphotropic virus (HTLV)–associated myelopathy/tropical spastic paraparesis (HAM/TSP); and 10 cases of HIV infection. All HIV-positive participants were on antiretroviral therapy and had no other neurological comorbidities.
- NIND category comprises 43 individuals with small vessel disease or migraine.

In the first test set from Verona:

- OIND category comprises 2 cases of neuromyelitis optica spectrum disorder (NMOSD; 2 AQP4 antibody–positive); 10 cases of myelin oligodendrocyte glycoprotein antibody–associated disease (MOGAD); 2 cases of Behçet disease, 1 case of lymphoma.
- NIND category comprises 3 individuals with small vessel disease or migraine.

**1.3. Image processing and biomarker assessment**

Data were collected as DICOM images and processed using MIPAV (Medical Image Processing, Analysis & Visualization, NIH) and BMAT^7^ (https://github.com/BMAT-Apps). The CVS assessment was performed on FLAIR* images generated by coregistration and voxel-wise multiplication of the high-resolution 3D T2*-EPI magnitude and the 3D T2-FLAIR (https://github.com/BMAT-Apps/flairstar), as previously described,^8,9^ and the presence/absence of the CVS was assessed according to the NAIMS guidelines.^10^ For the Select3* algorithm,^11,12^ the scan was considered as CVS-positive if there were ≥ 3 eligible lesions that met the CVS-positive NAIMS criteria,^10^ or, when there were < 3 CVS-positive lesions, if CVS-positive lesions outnumbered CVS-negative lesions (2/3 CVS-positive/eligible lesions). If neither condition was met, the scan was rated as Select3*-negative. For the Select6* algorithm,^12,13^ each scan was considered as CVS-positive if there were ≥ 6 eligible CVS-positive lesions or, when there were < 6 CVS-positive lesions, if CVS-positive lesions outnumbered CVS-negative lesions. If neither condition was met, the scan was rated as Select6*-negative. MRI scans with < 3 lesions eligible for the CVS assessment were considered as “not applicable” for the analysis for both Select3* and Select6*.

CL presence was determined on DIR images as hyperintensity compared to adjacent normal-appearing grey matter and confirmed on MPRAGE sequences as hypointense signal relative to the surrounding normal cortex, following previously published guidelines.^5,14^ When DIR sequences were not available (centers other than Brussels), CL were detected on synthetic-DIR generated from T2/T1 images.^4^ Synthetic-DIR images were obtained by dividing preprocessed (N4 bias correction^15^ and denoising^16^) 3D-FLAIR by the 3D-MPRAGE images for each subject. Confluent lesions extending from white matter and leukocortical lesions < 3mm in diameter were excluded.

PRL were identified on 3D T2*-EPI unwrapped phase images (<https://github.com/BMAT-Apps/phase_unwrap>)^7^ as chronic lesions showing a hypointense rim and internal isointensity to extralesional white matter, and without enhancement on post-contrast T1-weighted sequences.^17,18^

In each patient, CVS, CL, and PRL analysis was independently assessed following established guidelines and methods by 2 trained investigators (P.M. and S.B. for Erasme, CUSL, CHUV and Verona cases; M.A. and M.S.M. for Milan and NIH cases), each unaware of the other's analysis and blinded to participant's diagnosis. All non-MS cases that were CVS-positive or bearing ≥1 PRL or ≥1 CL were jointly reviewed by M.A. and P.M. for final adjudication.

**1.4. Machine Learning Framework and Evaluation Protocol**

This section outlines the approach employed to investigate the application of ML in diagnosing MS using only cross-sectional demographic and radiological data. The diagnostic task was formulated as a binary classification problem, using covariates detailed in Section 2.1 to predict MS diagnosis. The first objective (Step 1) was to identify all 71 possible optimal algorithm-combination sets. Algorithm-combination sets refer to pairings of ML algorithms with input covariate combinations from different biomarker categories (e.g., %CVS+CL1+DIS, #CL+PRL1, etc.) that optimize diagnostic accuracy. Step 2 evaluated these pairs against the classification performance of DIS according to McDonald 2017 criteria (hereafter referred as “the baseline”) and ranked them by their relative improvement in diagnostic accuracy. The highest-performing algorithm-combination pair was then compared against all other pairs (step 3), followed by a similar analysis restricted to models using only simplified assessed covariates (Step 4). Step 5 analysed the performance difference between these two models. Finally, a generalization study evaluates robustness of our best performing algorithm-combination on unseen data. The code for all experiments is available on GitHub (<https://github.com/maxencewynen/MS-Diagnostic-Tool-UCLouvain>.

**1.4.1. Task definition**

The automatic diagnostic issue, framed as a classification task, necessitates the definition of four components: output, input, ML algorithm, and performance metric. The binary output designates MS presence (1) or absence (0). Input covariates initially encompassed demographic and radiological information: *Age*, *Sex assigned at birth*, *DIS*, *%CVS*, *Select-3/6**, *#CL*, *CL1*, *#PRL*, *PRL1*, as detailed in Section 2.1. Following statistical analysis, ANOVA F-value feature selection excluded *Age* and *Sex assigned at birth* due to the insufficient discriminatory power of these variables (p < 0.05). To address missing biomarker evaluation, separate ML models (see here below) were trained for each variable combination, ensuring that no combination included the same variable/biomarker at different levels (e.g., *#PRL* and *PRL1* in the same model). This yielded to 71 distinct variable combinations.

The ML algorithms selected for this study include logistic regression^19^ (LR), decision tree,^20^ random forest (RF),^21^ K-nearest neighbours classifier (KNN),^22^ support vector classifier (SVC),^23^ and extreme gradient boosting (XGB).^24^ These algorithms were chosen due to their widespread use, demonstrated performance, and accessibility via python libraries. Finally, *balanced accuracy* — defined as the average of specificity and sensitivity — was selected as the performance metric to be maximized. This metric was chosen to ensure an optimal trade-off between sensitivity and specificity, thereby providing a robust evaluation of model performance.

**1.4.2. Dataset partitioning**

Prior to conducting any analyses, the dataset was partitioned into a training set, comprising data from five centres (n = 285), and two independent test sets (n = 79), selected specifically to evaluate the generalisability of the ML models to unseen and heterogeneous data (see Table 1). The first test set included data from Verona, Italy (n = 37) to ensure scanner and geographical diversity. The second test set included prodromal cases from CUSL (n = 84) to test the models against the upcoming 2024 McDonald criteria for early diagnosis of MS.

**1.4.3. Identification of Optimal Algorithm-Combination Pairs (Step 1)**

Step 1 identified optimal algorithm-combination pairs by determining the ML algorithm that maximized performance for each of the 71 variable combinations. Using 10-fold cross-validation (CV) on the training set, the algorithm with the highest balanced accuracy was selected for each combination. The optimal cutoff for classifying the output as positive (MS) or negative (not MS) was determined by averaging the best-performing cutoffs across all folds, defined as the value maximizing balanced accuracy.

**1.3.4. Algorithm-Combination Pairs Evaluation and Comparison (Steps 2-5)**

Steps 2-5 were conducted exclusively on the training set. Step 2 assessed all algorithm-combination pairs against the baseline using the 5x2CV combined F-test,^25,26^ a statistical method that applies five iterations of 2-fold CV to compute an F-statistic for significance testing. This analysis had two objectives: (1) categorizing pairs into those that significantly outperformed the baseline (p < 0.05) and those that did not, and (2) ranking pairs based on their relative improvement in balanced accuracy over the baseline. The best overall algorithm-combination pair (*M_Best_*) and the best pair that used only simplified variables (*M_Simplified_*) were identified.

In step 3, *M_Best_* was compared to all other pairs to determine whether its superiority was statistically significant, using the same statistical method and level of significance. Step 4 applied this approach to compare *M_Simplified_* against all other pairs that also used only simplified variables as input. Finally, step 5 specifically compared *M_Best_* against *M_Simplified_*, again employing the 5x2CV framework for significance testing.

**1.4.5. Generalisability Assessment**

To ensure model robustness and generalisability, performance was evaluated on independent, unseen datasets, mitigating the risk of overfitting. All models, but specifically *M_Best_* and *M_Simplified_*, were retrained on the full training set, incorporating data from all five centres, to maximize available information. All models’ performance was then assessed on the two independent test sets and compared to the baseline DIS performance by conducting a holistic evaluation using balanced accuracy, sensitivity, specificity, precision, and F1 score metrics. Finally, we identified *M_Generaliser_*, a model outperforming DIS on the training set that provides the highest performance on both test sets.

**1.5. Online diagnostic tool**

Accompanying this paper, the authors have made available an open-access online tool (see Figure 3, [https://www.msdiagnostictool.org](https://www.msdiagnostictool.org/)) following a MDCalc fashion, which enables users to input patient data and instantly receive diagnostic prediction from the study's models, facilitating direct application of the research findings and reproducibility of the results. For each model, additional interpretability and explainability plots are provided.^27^

To ensure the reliability and interpretability of the models deployed on the website, several measures were implemented, including model calibration, uncertainty quantification, user-friendly output presentation, and SHAP (SHapley Additive exPlanations)-based explainability.^27^

Since the raw outputs of classifiers often do not represent true posterior probabilities,^28^ model calibration is essential to adjust predicted probabilities to better reflect actual likelihoods, enabling reliable interpretation and comparison of different model outputs. Platt’s calibration method from scikit-learn was used with a 5-fold CV, which has been shown to perform well on small datasets,^28,29^ thus producing five distinct calibrated models for each algorithm-combination pair. The final output displayed on the website is the average of these predictions, accompanied by their standard deviation, providing both a robust estimate and a measure of uncertainty of the algorithm-combination pair.

Next, to mitigate potential confusion arising from displaying the outputs of up to 71 models, we implemented three summarized visual aids (Figure 3). The first visual aid (Fig 3a) presents the output of the best-performing model — defined as the algorithm-combination pair that showed the best performance across the two test sets — alongside a summary of predictions from all available models, separated between models that significantly outperformed DIS on the training dataset, and those that did not. Additionally, a summary chart (Fig 3b) displays the outputs of all models, sorted by their expected performance on the test sets, while a summary table provides detailed information about each model, including its algorithm, input combination, and key performance metrics. This presentation allows users to compare predictions across models and evaluate their consistency.

Finally, to enhance explainability, we employed SHAP values,^27^ a method based on cooperative game theory. This method decomposes the model’s predictions into the contributions of individual features, enabling users to understand how specific variables influence the output. This transparency ensures that the model’s predictions are interpretable and actionable. By ensuring transparency and enabling users to understand the models’ decision-making process, the four measures we developed in this section support a practical application of the proposed tool in clinical settings.

1. Supplementary results

**Supplementary Table 2**. Diagnostic performance of machine learning models significantly outperforming the baseline dissemination in space (DIS) criterion. The table summarizes the input variables, algorithm, assessment type (Full-count or Simplified), balanced accuracy, improvement in balanced accuracy over baseline with 95% confidence interval (CI) and statistical significance (p-value and p-value corrected for multiple comparison by applying a Benjamini–Hochberg false discovery rate (FDR) correction), and sensitivity and specificity for each model. Abbreviations: dissemination in space (DIS), XGBoost classifier (XGB), support vector machine classifier (SVC), central vein sign (CVS), cortical lesions (CL), paramagnetic rim lesions (PRL).

| **Input variables** | **Model** | **Assessment** | **Balanced accuracy** **(%)** | **Difference with DIS** **(%) [95% CI]** | **p-value/FDR p-value** | **Sensitivity (%)** | **Specificity (%)** |
| --- | --- | --- | --- | --- | --- | --- | --- |
| **DIS (Baseline)** | **-** | **-** | 82.8 | **-** | **-** | 100 | 65.4 |
| %CVS, #CL, #PRL | Random Forest | Full-count | 95.7 | +13.0 [+10.5; +17.0] | .00198/.00541 | 97.5 | 93.4 |
| %CVS, #CL, PRL1 | Random Forest | Full-count | 95.7 | +13.0 [+10.5; +17.1] | .00191/.00541 | 97.5 | 92.9 |
| %CVS, CL1 | Logistic Regression | Full-count | 95.6 | +12.9 [+8.7; +16.7] | .00618/.01185 | 97.8 | 93.3 |
| %CVS, CL1, PRL1 | Logistic Regression | Full-count | 95.5 | +12.8 [+8.5; +16.0] | .00407/.00876 | 97.5 | 93.5 |
| DIS, %CVS, CL1 | Logistic Regression | Full-count | 95.5 | +12.8 [+8.8; +16.6] | .00449/.00938 | 98.2 | 92.8 |
| DIS, %CVS, CL1, PRL1 | Logistic Regression | Full-count | 95.5 | +12.8 [+8.6; +16.0] | .00374/.00830 | 97.8 | 93.1 |
| DIS, %CVS, PRL1 | XGB | Full-count | 95.4 | +12.7 [+9.4; +16.0] | .00221/.00541 | 98.2 | 92.6 |
| DIS, %CVS, CL1, #PRL | Logistic Regression | Full-count | 95.3 | +12.6 [+8.5; +16.3] | .00564/.01113 | 97.5 | 93.1 |
| DIS, %CVS, #PRL | Logistic Regression | Full-count | 95.3 | +12.6 [+9.1; +15.3] | .00129/.00510 | 98.5 | 92.1 |
| %CVS, #PRL | Logistic Regression | Full-count | 95.1 | +12.4 [+9.1; +15.2] | .00202/.00541 | 98.5 | 91.8 |
| %CVS, CL1, #PRL | Logistic Regression | Full-count | 95.1 | +12.4 [+8.5; +16.3] | .00782/.01292 | 97.3 | 92.9 |
| %CVS, #CL | Random Forest | Full-count | 94.8 | +12.1 [+7.9; +17.5] | .00668/.01201 | 97.3 | 94.4 |
| DIS, %CVS, #CL, #PRL | Random Forest | Full-count | 94.8 | +12.1 [+8.6; +15.9] | .00174/.00541 | 97.5 | 97.1 |
| DIS, %CVS | Random Forest | Full-count | 94.6 | +11.9 [+9.2; +14.9] | .00098/.00510 | 98.8 | 90.1 |
| %CVS | Random Forest | Full-count | 94.6 | +11.9 [+7.2; +15.8] | .00677/.01201 | 98.7 | 90.3 |
| DIS, %CVS, #CL, PRL1 | Random Forest | Full-count | 94.5 | +11.8 [+6.5; +15.9] | .00291/.00667 | 97.9 | 91.5 |
| %CVS, PRL1 | Random Forest | Full-count | 94.4 | +11.7 [+8.4; +15.8] | .00467/.00947 | 97.8 | 90.9 |
| DIS, %CVS, #CL | Random Forest | Full-count | 94.2 | +11.5 [+7.2; +14.8] | .00192/.00541 | 97.6 | 91.7 |
| Select3*, CL1, PRL1 | Logistic Regression | Simplified | 94.1 | +11.4 [+7.7; +13.3] | .00122/.00510 | 96.2 | 92.0 |
| Select3*, CL1, #PRL | Logistic Regression | Full-count | 94 | +11.3 [+9.1; +13.1] | .00029/.00426 | 95.9 | 92.1 |
| DIS, Select3*, #CL | Random Forest | Full-count | 93.5 | +10.8 [+8.9; +13.8] | .00058/.00482 | 96.3 | 90.5 |
| Select6*, CL1, PRL1 | Logistic Regression | Simplified | 93.3 | +10.6 [+6.4; +13.8] | .00757/.01279 | 95.2 | 91.5 |
| DIS, Select3*, #CL, #PRL | Random Forest | Full-count | 93.3 | +10.6 [+7.1; +14.4] | .00137/.00510 | 95.5 | 91.3 |
| Select3*, #CL, PRL1 | SVC | Full-count | 93 | +10.3 [+8.1; +12.3] | .00123/.00510 | 93.7 | 91.4 |
| Select6*, CL1, #PRL | Logistic Regression | Full-count | 92.9 | +10.2 [+6.0; +13.2] | .00707/.01224 | 94.7 | 91.2 |
| DIS, Select3*, #CL, PRL1 | Random Forest | Full-count | 92.9 | +10.2 [+7.1; +14.3] | .00178/.00541 | 95.6 | 90.6 |
| DIS, Select3*, CL1 | Random Forest | Simplified | 92.8 | +10.1 [+8.7; +12.4] | .00031/.00426 | 96.9 | 88.4 |
| DIS, Select3*, #PRL | Logistic Regression | Full-count | 92.7 | +10.0 [+8.1; +12.2] | .00107/.00510 | 97.2 | 88.3 |
| Select6*, #CL, PRL1 | Random Forest | Full-count | 92.6 | +9.9 [+6.0; +13.7] | .00213/.00541 | 92.1 | 93.0 |
| DIS, Select3*, PRL1 | Logistic Regression | Simplified | 92.4 | +9.7 [+7.9; +11.8] | .00061/.00482 | 98.0 | 86.8 |
| DIS, Select6*, #CL, #PRL | XGB | Full-count | 92.4 | +9.7 [+7.3; +12.3] | .00042/.00426 | 95.7 | 89.1 |
| DIS, Select6*, CL1 | Logistic Regression | Simplified | 92.4 | +9.7 [+8.1; +11.6] | .00081/.00510 | 98.0 | 86.8 |
| DIS, Select6*, #CL, PRL1 | XGB | Full-count | 92.3 | +9.6 [+7.3; +12.3] | .00074/.00510 | 95.7 | 88.9 |
| DIS, Select3*, CL1, #PRL | Logistic Regression | Full-count | 92.2 | +9.5 [+7.8; +12.2] | .00149/.00530 | 98.2 | 86.3 |
| DIS, Select6*, PRL1 | Decision Tree | Simplified | 92.2 | +9.5 [+8.1; +10.9] | .00021/.00426 | 96.1 | 88.2 |
| DIS, Select6*, CL1, #PRL | XGB | Full-count | 92.2 | +9.5 [+6.3; +13.4] | .00220/.00541 | 95.2 | 88.1 |
| Select6*, #CL, #PRL | Random Forest | Full-count | 91.9 | +9.2 [+4.1; +13.4] | .00253/.00599 | 92.8 | 92.0 |
| DIS, Select3*, CL1, PRL1 | Logistic Regression | Simplified | 91.8 | +9.1 [+8.2; +11.1] | .00012/.00413 | 98.9 | 84.7 |
| DIS, Select6*, #CL | SVC | Full-count | 91.7 | +9.0 [+7.1; +11.3] | .00115/.00510 | 97.1 | 88.8 |
| Select3*, #CL, #PRL | SVC | Full-count | 91.6 | +8.9 [+6.4; +12.4] | .00667/.01201 | 92.2 | 90.9 |
| DIS, Select6*, #PRL | Logistic Regression | Full-count | 91.5 | +8.8 [+7.4; +10.5] | .00040/.00426 | 96.7 | 86.3 |
| DIS, Select6*, CL1, PRL1 | Logistic Regression | Simplified | 91.4 | +8.7 [+6.7; +10.9] | .00121/.00510 | 98.2 | 84.6 |
| Select3*, #CL | Random Forest | Full-count | 90.4 | +7.7 [+3.2; +11.7] | .02007/.03115 |  |  |
| Select6*, #PRL | XGB | Full-count | 89.7 | +7.0 [+1.9; +10.1] | .02018/.03115 | 90.4 | 89.0 |
| Select6*, PRL1 | Logistic Regression | Simplified | 89.2 | +6.5 [+1.6; +9.8] | .03484/.04947 | 90.5 | 87.9 |
| DIS, Select3* | Logistic Regression | Simplified | 89.2 | +6.5 [+0.0; +12.7] | .01674/.02702 | 97.4 | 80.9 |
| CL1, #PRL | SVC | Full-count | 88.9 | +6.2 [+3.5; +9.5] | .03855/.05366 |  |  |
| Select3*, #PRL | SVC | Full-count | 88.7 | +6.0 [+2.9; +9.3] | .04191/.05722 |  |  |
| Select3*, PRL1 | Logistic Regression | Simplified | 88.6 | +5.9 [+3.2; +9.4] | .03293/.04772 | 96.5 | 80.6 |
| Select3* | Logistic Regression | Simplified | 88.4 | +5.7 [+3.2; +8.6] | .02200/.03324 | 97.2 | 79.7 |
| #CL, #PRL | XGB | Full-count | 88.1 | +5.3 [+2.6; +8.8] | .02923/.04323 | 89.5 | 86.6 |

**Supplementary Table 3**. Pairwise comparison of model performance relative to M_Best_ and M_Simplified_ assessed on test set 2 (Prodromal dataset). The table reports the input variables, algorithm, and balanced accuracy with 95% confidence intervals (CI) for each model, along with the results of one-sided Wilcoxon signed-rank tests assessing whether the balanced accuracy of either M_Best_ or M_Simplified_ was significantly greater than that of the corresponding model. Reported p-values correspond to the hypotheses H₁: M_Best_ > Model and H₁: M_Simplified_ > Model, respectively. Abbreviations: central vein sign (CVS), cortical lesions (CL), paramagnetic rim lesions (PRL).

| Model | Input Variables | Balanced Accuracy  (%) [CI] | *p*(*M_Best_*  > Model) | *p*(*M_Simplified_* > Model |
| --- | --- | --- | --- | --- |
| *M_Best_* | *%CVS, #PRL, #CL* | 93.9 [88.1; 98.8] | - | - |
| *M_Simplified_* | *Select-3*, CL1, PRL1* | 92.6 [86.3; 97.6] | - | - |
| Random Forest Classifier | *%CVS* | 88.9 [81.8; 95.1] | p < 0.001 | p < 0.001 |
| - | *Select-3** | 91.3 [84.8; 97.4] | p < 0.001 | p < 0.001 |
| - | *Select-6** | 92.6 [56.5; 97.7] | p < 0.001 | p < 0.001 |
| Logistic Regression | *#CL* | 73.1 [63.3; 82.5] | p < 0.001 | p < 0.001 |
| - | *CL1* | 73.2 [63.2; 82.5] | p < 0.001 | p < 0.001 |
| Random Forest Classifier | *#PRL* | 74.9 [66.9; 83.0] | p < 0.001 | p < 0.001 |
| - | *PRL1* | 74.9 [66.4; 83.4] | p < 0.001 | p < 0.001 |

Supplementary references :

1. Absinta, M. *et al.* Identification of Chronic Active Multiple Sclerosis Lesions on 3T MRI. *Am. J. Neuroradiol.* **39**, 1233–1238 (2018).

2. Sati, P., George, I. C., Shea, C. D., Gaitán, M. I. & Reich, D. S. FLAIR*: A Combined MR Contrast Technique for Visualizing White Matter Lesions and Parenchymal Veins. *Radiology* **265**, 926–932 (2012).

3. Sati, P. *et al.* Rapid, high-resolution, whole-brain, susceptibility-based MRI of multiple sclerosis. *Mult. Scler. Houndmills Basingstoke Engl.* **20**, 1464–1470 (2014).

4. Manning, A. R. *et al.* T1/T2 ratio from 3T MRI improves multiple sclerosis cortical lesion contrast. *J. Neuroimaging* **33**, 434–445 (2023).

5. Filippi, M. *et al.* Assessment of lesions on magnetic resonance imaging in multiple sclerosis: practical guidelines. *Brain J. Neurol.* **142**, 1858–1875 (2019).

6. Kober, T. *et al.* MP2RAGE Multiple Sclerosis Magnetic Resonance Imaging at 3 T. *Invest. Radiol.* **47**, 346 (2012).

7. Vanden Bulcke, C. *et al.* BMAT: An open-source BIDS managing and analysis tool. *NeuroImage Clin.* **36**, 103252 (2022).

8. Maggi, P. *et al.* Central vein sign differentiates Multiple Sclerosis from central nervous system inflammatory vasculopathies. *Ann. Neurol.* **83**, 283–294 (2018).

9. Maggi, P. *et al.* The ‘central vein sign’ in patients with diagnostic ‘red flags’ for multiple sclerosis: A prospective multicenter 3T study. *Mult. Scler. Houndmills Basingstoke Engl.* **26**, 421–432 (2020).

10. Sati, P. *et al.* The central vein sign and its clinical evaluation for the diagnosis of multiple sclerosis: a consensus statement from the North American Imaging in Multiple Sclerosis Cooperative. *Nat. Rev. Neurol.* **12**, 714–722 (2016).

11. Solomon, A. J. *et al.* Diagnostic performance of central vein sign for multiple sclerosis with a simplified three-lesion algorithm. *Mult. Scler. Houndmills Basingstoke Engl.* **24**, 750–757 (2018).

12. Ontaneda, D. *et al.* Central vein sign: A diagnostic biomarker in multiple sclerosis (CAVS-MS) study protocol for a prospective multicenter trial. *NeuroImage Clin.* **32**, 102834 (2021).

13. Mistry, N. *et al.* Imaging central veins in brain lesions with 3-T T2*-weighted magnetic resonance imaging differentiates multiple sclerosis from microangiopathic brain lesions. *Mult. Scler. Houndmills Basingstoke Engl.* **22**, 1289–1296 (2016).

14. Geurts, J. J. G. *et al.* Consensus recommendations for MS cortical lesion scoring using double inversion recovery MRI. *Neurology* **76**, 418–424 (2011).

15. Tustison, N. J. *et al.* N4ITK: Improved N3 Bias Correction. *IEEE Trans. Med. Imaging* **29**, 1310–1320 (2010).

16. Manjón, J. V., Coupé, P., Martí-Bonmatí, L., Collins, D. L. & Robles, M. Adaptive non-local means denoising of MR images with spatially varying noise levels. *J. Magn. Reson. Imaging* **31**, 192–203 (2010).

17. Yao, B. *et al.* Chronic multiple sclerosis lesions: characterization with high-field-strength MR imaging. *Radiology* **262**, 206–215 (2012).

18. Martire, M. S., Moiola, L., Rocca, M. A., Filippi, M. & Absinta, M. What is the potential of paramagnetic rim lesions as diagnostic indicators in multiple sclerosis? *Expert Rev. Neurother.* **22**, 829–837 (2022).

19. Freund, Y. & Schapire, R. E. A Decision-Theoretic Generalization of On-Line Learning and an Application to Boosting. *J. Comput. Syst. Sci.* **55**, 119–139 (1997).

20. Quinlan, J. R. Induction of decision trees. *Mach. Learn.* **1**, 81–106 (1986).

21. Breiman, L. Random Forests. *Mach. Learn.* **45**, 5–32 (2001).

22. KNeighborsClassifier. *scikit-learn* https://scikit-learn/stable/modules/generated/sklearn.neighbors.KNeighborsClassifier.html.

23. Cortes, C. & Vapnik, V. Support-vector networks. *Mach. Learn.* **20**, 273–297 (1995).

24. XGBoost | Proceedings of the 22nd ACM SIGKDD International Conference on Knowledge Discovery and Data Mining. *ACM Conferences* https://dl.acm.org/doi/10.1145/2939672.2939785 doi:10.1145/2939672.2939785.

25. Alpaydin, E. Combined 5 x 2 cv F test for comparing supervised classification learning algorithms. *Neural Comput.* **11**, 1885–1892 (1999).

26. Raschka, S. Model Evaluation, Model Selection, and Algorithm Selection in Machine Learning. Preprint at https://doi.org/10.48550/arXiv.1811.12808 (2020).

27. Lundberg, S. M. & Lee, S.-I. A Unified Approach to Interpreting Model Predictions. in *Advances in Neural Information Processing Systems* (eds. Guyon, I. et al.) vol. 30 (Curran Associates, Inc., 2017).

28. Predicting good probabilities with supervised learning | Proceedings of the 22nd international conference on Machine learning. *ACM Other conferences* https://dl.acm.org/doi/10.1145/1102351.1102430 doi:10.1145/1102351.1102430.

29. Platt, J. Probabilistic Outputs for Support vector Machines and Comparisons to Regularized Likelihood Methods. in (1999).
